# Tranverese Myelitits and Myelography

**Published:** 1986-06

**Authors:** Peter A. Rowe, W. Peter Gorman

**Affiliations:** Dept. of Medicine, Southmead General Hospital, Bristol (current appointment: Clinical Research Fellow, Dept. of Renal Medicine, City Hospital, Nottingham NG5 1PB); Dept. of Medicine, Southmead General Hospital, Bristol (current appointment: Senior Registrar, Dept. of Medicine, Royal Hallamshire Hospital, Sheffield S10 2JF)

**Keywords:** transverse myelitis, optic neuritis, myelography

## Abstract

A case report of transverse myelitis and optic neuritis is presented in which the latter occurred following a myelogram with iopamidol. The literature relating to neurotoxicity of contrast agents is reviewed, and it is suggested that the optic neuritis may have been a neurotoxic effect of iopamidol.


### Transverse myelitis and myelography

**Peter A. Rowe M.R.C.P.**
Dept. of Medicine, Southmead General Hospital, Bristol (current appointment: Clinical Research Fellow, Dept. of Renal Medicine, City Hospital, Nottingham NG5 1PB)
**W. Peter Gorman M.R.C.P.**
Dept. of Medicine, Southmead General Hospital, Bristol (current appointment: Senior Registrar, Dept. of Medicine, Royal Hallamshire Hospital, Sheffield S10 2JF)

### ABSTRACT

A case report of transverse myelitis and optic neuritis is presented in which the latter occurred following a myelogram with iopamidol. The literature relating to neurotoxicity of contrast agents is reviewed, and it is suggested that the optic neuritis may have been a neurotoxic effect of iopamidol.

### KEY WORDS

transverse myelitis, optic neuritis, myelography.

### INTRODUCTION

In a patient with an acute spinal cord syndrome it may be difficult to distinguish clinically between cord compression and a transverse myelitis (1). To exclude a compressive lesion, myelography, although an important investigation, is not without risk. Adverse effects such as the complications of lumbar puncture are well known but other, less familiar, reactions occur due to neurotoxicity of the contrast agent. We report a case of transverse myelitis in which optic neuritis followed myelography with iopamidol (Niopam 300).

### CASE REPORT

A 19 year old woman developed an upper respiratory tract infection and two weeks later presented with progressive weakness and paraesthesiae of the lower limbs and acute retention of urine. She denied back pain, headache and visual symptoms. At the age of 2 years she had suffered from an episode of lead poisoning and at 18 years complained of excessive somnolence and poor concentration; at that time a computed tomographic (CT) brain scan and EEG were normal.

Examination revealed normal speech and higher mental function. There was pyramidal weakness in the legs (grade 4 MRC scale). Reflexes were brisk and symmetrical with flexor plantar responses, and tone normal. Appreciation of pain and light touch was reduced in the T10 to L2 dermatomes. Anal tone was diminished. Bladder catheterisation produced a residual volume of 900 mls.

A structural spinal cord lesion was suspected but myelography of the entire spinal cord, using 8 mls of iopamidol (Niopam 300) via a lumbar approach, showed no abnormalities. Cerebrospinal fluid (CSF) analysis showed 240 lymphocytes/l with normal glucose and protein. The next day she had back pain and meningism, and developed a paraplegia over the following 2 days. On the fourth day she complained of blurred vision, which progressed within 12 hours to complete blindness due to papillitis. A further CT brain scan was normal. Dexamethasone was given (4 mg thrice daily). Repeat CSF examination revealed an increased lymphocyte count (650/1), elevated protein (2410 mg/l) and normal glucose. An extensive microbiological screen for possible underlying bacterial, viral or fungal infection was negative. An autoantibody screen was negative (including antinuclear factor), complement levels were normal, and tests for circulating immune complexes gave equivocal results. CSF IgG/total protein ratio was 12% (normal), and CSF cytology unhelpful. Nerve conduction studies were compatible with a mixed upper and lower motor neuropathy. When visual acuity had returned to normal (N5) three weeks after admission, visual evoked responses were poorly formed and significantly delayed (145 msec bilaterally, normal range 88–112 msec). After 18 months a complete flaccid paraplegia remains with extensor plantar responses and a sensory level at T5, and continuing urinary retention is managed by intermittent self-catheterisation.

### COMMENT

Our patient presented with a spinal cord lesion probably due to transverse myelitis. The precise diagnosis is open to debate as there is considerable overlap between the various neuropathological causes of this clinical picture. Transverse myelitis often follows a variety of bacterial, viral and protozoal infections (2), and would best fit the clinical course in this case, but would not explain the optic neuritis. The combination of transverse myelitis and optic neuritis has been called neuromyelitis optica or Devic's disease (3). This is, however, a heterogenous condition which Cloys and Netsky (4) regard as a purely clinical syndrome of varied aetiology. CSF findings are usually of a polymorphonuclear leukocytosis and elevated protein level. Other possible diagnoses include multiple sclerosis – although the time course of a single episode, without disturbance of CSF immunoglobulins, makes this less likely – or an adverse reaction to the radiological contrast agent.

Myelography is associated with a number of neurotoxic effects including meningism, arachnoiditis, myelopathy, seizures, sixth cranial nerve palsies and cognitive disturbance (5). Visual disturbances have been described with oil (6) and water-soluble (7) contrast media but not with iopamidol (Committee on Safety of Medicines – personal communication). Non-ionic water-soluble media with a low osmolality such as iopamidol are reportedly associated with fewer adverse reactions (8). Nevertheless, these agents diffuse into the intracranial CSF compartment even against gravity, and can penetrate the brain parenchyma (9,10). Neurotoxic effects are more common when a large volume of undiluted contrast agent is given, as in this case to visualise the whole spinal cord, and minor adverse effects are more frequent

*(continued on page 59)*

Correspondence to Dr. Peter A. Rowe, Dept. of Renal Medicine, City Hospital, Hucknall Road, Nottingham NG5 1PB.

### Transverse myelitis and myelography *(continued from page 58)*

in patients with multiple sclerosis (11), another demyelinating condition.

Although further evolution of the clinical picture following presentation was not unexpected, we suggest that the myelogram may have exacerbated the course of her disease, and speculate that the optic neuritis may have been a neurotoxic effect. We report this interesting case to highlight the difficulty of establishing a precise neuropathological diagnosis, and to remind clinicians of the potential hazards associated with myelography.

### REFERENCES

1. SCHAFER, ER. (1975) The Spinal Compression Syndrome: A Review of Differential Diagnosis. In: Vinken PJ and Bruyn GW, (eds.) Handbook of Clinical Neurology. Oxford. North Holland Publishing. **19**, pp. 347–386.
2. BERMAN, M. FELDMAN, S. ALLER, M. ZILBER, N. and KAHANA, E. (1981) Acute transverse myelitis: Incidence and etiologic considerations. Neurology (NY). **31**, pp. 966–971.
3. GAULT, F. (1894) De la neuromyelite optique aigue. Thesis, Lyon.
4. CLOYS, DE. and NETSKY, MG. (1982) Neuromyelitis Optica. In: Vinken PJ and Bruyn GW, (eds.) Handbook of Clinical Neurology. Oxford. North Holland Publishing. **9**, pp. 426–436.
5. JUNCK, L. MARSHALL, WH. (1983) Neurotoxicity of radiological contrast agents. Annals of Neurology. **13**, pp. 469–484.
6 CRISTI, G. SCIALFA G. DI PIERRO, G. and TASSONI, A. (1974) Visual loss: A rare complication following oil myelography. Neuroradiology. **7**, pp. 287–290.
7 LEAVENGOOD, JM. WILSON, WB. STEARS, J. and POSNER, JB. (1981) Reversible visual deficits following metrizamide (Amipaque) myelography. Neurology (NY). **31**, p. 69.
8. BASSI, P. CECCHINI, A. DETTORI, P. and SIGNORINI, E. (1982) Myelography with Iopamidol, a non-ionic water soluble contrast medium: incidence of complications. Neuroradiology. **24**, pp. 85–90.
9. SMIRNIOTOPOULOS, JG. MURPHY, FM. SCHELLINGER, D. KURTYKE, JF. and BORTS, FT. (1984) Cortical blindness after metrizamide myelography. Arch. Neurol. **41**, pp. 224–226.
10. DRAYER, BP. and ROSENBAUM, AE. (1977) Metrizamide brain penetrance. Acta. Radiol. (suppl) (Stockholm). **355**, pp. 280–293.
11. KAUFMAN, P. and JEANS, WD. (1976) Reactions to Iophendylate in relation to multiple sclerosis. Lancet. **2**, pp.1000–1001.

